# Identification of the socio-cultural barriers of drug addiction treatment in Iran

**DOI:** 10.1016/j.heliyon.2023.e15566

**Published:** 2023-04-18

**Authors:** Emran Razaghi, Ali Farhoudian, Azam Pilevari, Alireza Noroozi, Zahra Hooshyari, Ramin Radfar, Mohsen Malekinejad

**Affiliations:** a Department of Psychiatry, Roozbeh Hospital, Tehran University of Medical Sciences, Tehran, Iran; b Department of Sociology, Kharazmi University, Karaj, Iran; c Iranian National Center for Addiction Studies (INCAS), Tehran University of Medical Science, Tehran, Iran; d Department of Geriatric Medicine, Ziaeian Hospital, Tehran University of Medical Sciences (TUMS), Tehran, Iran; e Medical Education and Management, Shahid Beheshti University of Medical Sciences, Tehran, Iran; f Department of Neuroscience and Addiction, School of Advanced Technologies in Medicine (SATiM), Tehran University of Medical Sciences, Tehran, Iran; g Department of Epidemiology and Biostatistics, School of Medicine, University of California, San Francisco, USA

**Keywords:** Qualitative study, Barriers, Substance-related disorders, Treatment

## Abstract

**Introduction:**

Socio-cultural norms can either be encouraging or a barrier to addiction treatment. More, rigorous research is needed on nonindigenous models in addiction treatment, to better understand the role of socio-cultural differences.

**Methods:**

The present qualitative study is part of the project, “The Inclusive Assessment of the Barriers of Drug Addiction Treatment Services in Iran,” which was conducted in Tehran from 2018 to 2021. The participants consisted of eight people who used drugs, seven individual family members of the people who used drugs participants, seven service providers, and four policymakers. A purposeful sampling method was used for the selection of the participants, and the process continued until reaching the theoretical saturation of data. Analysis used the Graneheim and Lundman methods, classifying primary codes, the sub-themes, and themes were classified according to the similarities and differences between primary codes.

**Finding:**

The most important socio-cultural barriers to addiction treatment in Iran are: unrealistic expectations of the family and society from the people who use drugs, addiction stigma, mistrust between various components of the treatment system, perceptions that professional substance use disorder treatment is inefficient and low uptake of that treatment, the disturbed relational boundaries between the people who use drugs and their relatives, the interweaving of treatment and ethical and religious principles, low acceptance of maintenance treatments, treatment focusing on short-term outcomes, and presence of facilitating backgrounds of using drugs.

**Conclusions:**

The Iranian socio-cultural characteristics play an important role in the addiction treatment of the people who use drugs, so it is necessary for treatment interventions to be sensitive to these characteristics.

## 1 Introduction

Beliefs, cultural traditions, and social circumstances not only influence individuals’ behaviors (Center for Substance Abuse Treatment, 2006) but also form the basis of clinical services [1], welfare policies [2], family support (Center for Substance Abuse Treatment, 2006) for people who use drugs (PWUD), and workplace attitudes [3]. It has been shown that providing culturally-adapted substance use disorders (SUDs) treatment interventions is associated with increased treatment engagement and improved treatment outcomes [[4], [5], [6], [7], [8]] such as better relationships, more accurate diagnosis, positive therapeutic alliance, and higher client satisfaction [9].

Moreover, studies reveal that subjects such as stigma [10,11] faith, spirituality [12,13], ethnic and racial differences [[14], [15], [16]], the dominance of individualistic or collectivist mentalities in some cultures [17], regional attitudes towards using drugs [18] and turning to a special kind of drug or, even, a specific route of drug administration [19] are influenced by socio-cultural factors that lead to significant differences in treatment pathways. In societies where SUDs and PWUD are stigmatized, the PWUD are faced with more pressure to stop substance use which might translate to drug treatment demand [20].

Among these cultural and social elements, some believe that confidence in treatment influences not only treatment outcomes and patient-provider relationships [21], but also family cooperation with the treatment, and provider motivation and innovation [22,23], as well as abstinence from or entry in treatment environments [24].

A lack of trust entails more risks for PWUD receiving treatment services [24,25]. External attribution, fatalism, and the beliefs such as “if it is to happen, it happens,” are among other socio-cultural barriers that create barriers to behavior change for PWUD [26].

Prior studies of Latino and Asian populations found that when PWUD participants fulfilled family obligations or were able to manage family finances, it was less likely that they and their families would recognize problems related to using drugs [16,27,28]^.^ In such cases, families were observed responding to the substance use problem with ignorance, denial, and an expectation that the condition would self-resolve. The families in these studies accepted support only when they accepted that they were unable to solve or ignore the problem and could not preserve the family's reputation. However, they primarily preferred to seek help within the extended family, resorting to experts only when the addiction behavior became unbearable [27].

In addition, Asian families preferred to assign responsibilities to the PWUD, seeing their roles as unimportant in substance-use behavior change and looking for quick-fix solutions for the treatment due to disreputation [16,28].

Other socio-cultural factors affecting addiction treatment include: fear of miscommunication when receiving treatment not in one's mother tongue, PWUDs' fear of how their families may perceive them [16,29], doubts about providers' abilities [30], and the relationship of recovery to abstention [31].

In cultures where prevailing beliefs are that individual free will is sufficient to stop substance use [32], addiction behaviors are considered the result of PWUD irresponsibility. Accordingly, PWUD confront more judgments and stigma for receiving these services in these cultures [20] and are often obliged to observe stricter rules, such as perfect abstention or deprivation of freedom during the treatment period [24,33]. This leads to providers adopting a more severe approach to the PWUD [32]. In some cultures, including some Iranian ethnic groups (those people living in Xorasan, Kerman, East Azerbaijan, Kermanshah, Kurdistan, and Golestan), using opium and its derivates in ceremonies such as weddings and mourning is a tradition that has been associated with increased tendency to use drugs [34], and treatment demand might be negatively affected.

In societies, where there is more tolerance towards using drugs, there is less of a negative effect on PWUD's social life [35]. Beliefs encouraging using drugs, such as advising elders to use opium to promote their health and wellbeing [34], along with lack of explicit of condemnation of using some drugs in Islam despite the Islamic ban on alcohol use [36] are the among sociocultural factors that affect the initiation of drug use or demand for drug addiction treatment.

In Iranian traditional culture, using opium and its syrup are more socially accepted than using the drugs such as heroin and methamphetamine.

In this regard, Nyashanu & Visser (2022) state that cultural factors, especially where they are connected to using drugs or masculinity, severely reduce demand for treatment [37]. As mentioned, the complexity of addiction treatment necessitates the providers' recognition of socio-cultural factors that influences the recipients of their services. We believe that our inclusive and effective understanding of the reasons for receivers’ resistance to or nonobservance of the advice of service providers is related to this recognition.

Cultural sensitivities and recognition of the social realities of clients’ lives provide the necessary conditions for understanding the interpersonal dynamics, complications, and subtleties of interactions between service providers and clients, and leads to more effective commitment, flexibility, and effective interaction between them [24,38]. At present, few studies have dealt with certain cultural and social factors effective in the search or addiction treatment retention within various ethnicities or races [39]. Therefore, this study has been conducted with the goal of a more accurate understanding and recognition of certain socio-cultural factors effective on Iranian addiction treatment to answer the following two questions: How do Iranian socio-cultural characteristics reveal themselves in relations between providers and receivers of services, and How do these characteristics affect addiction treatment?

## 2 Methods

We conducted this research using the qualitative content analysis method. The participants in this study were from four groups including (a) PWUD referring to certified outpatient SUD treatment settings, (b) PWUDs' families, (c) service providers, and (d) policymakers in Tehran, all between 2018 and 2021. A purposeful sampling method was used for data collection. The authors, based on Glaser's approach (1978) referred to the samples which probably provided the maximum data [40]. These samples were often present in substance abuse treatment centers and mid-term residential centers, the two main pillars of treatment in Iran. To account, for the diversity among participants, we categorized the 22 districts of Tehran city into developed, semi-developed, and underdeveloped zones [41] and purposefully selected several SUD treatment centers from each zone (Table 1).

**Table 1:** The development level of Tehran's 22 districts.

District development zoning	Tehran's 22 districts
developed districts	1,2,3,5,6,7
moderately developed	4,8,11,13,21,22
underdeveloped zones	9,10,12,14,15,16,17,18,19,20

After the treatment centers were identified in each of these zones, the members of the research team selected three centers in developed zones, three centers in moderately developed zones, and five centers in the underdeveloped zone. Selection considered criteria such as flourishing or failing of the centers (taking into consideration the number of referrals), the familiarity of the unfamiliarity of these centers for service receivers and their cooperation with the research team.

Treatment team members recommended PWUD and family participants based on their ability to provide the most data and commentary. Participant selection also considered advice of PWUD and their families. Additional participants were sampled to fill information gaps.

The eligibility criteria for participants in PWUD and family member groups were (a) to provide informed consent, (b) to be age 16 years old or older, and (c) to be able to understand and answer questions.

Service providers were selected from among the managers and the treatment team members, taking into consideration factors such as a work history of more than 5 years and variety of disciplines. Policymakers were selected by the members of the research team using criteria such as a work history of at least 5 years in the treatment field or relevant policymaking and, preferably, variety in treatment approaches. The semi-structured interview was used as the data collection instrument. In order to prepare interview guidelines in local language, the existing literature was analyzed by two of the team members. They provided its primary draft. The draft provided was discussed by five members of the research team and finalized through two working sessions.

Data collection continued to theoretical enrichment. Eight PWUD and seven members of their families, seven service providers, and four policymakers were interviewed.

The interviews, which lasted for 45–120 min, began with a general inquiry on the subject of the study, such as “What are the barriers of SUD treatment in Iran?” and gradually focused on specific questions, such as, “what are either social or cultural barriers in the course of treatment?” If needed, exploratory questions were asked such as, “can you explain further?” or “can you give examples?”

Some of the interviews with service providers and policymakers’ groups were conducted virtually via Skype due to the COVID19 epidemic.

Data collection and analysis were done in parallel. After each interview, the audio file was transcribed verbatim. After coding each interview, the next one was performed. To analyze data, the conventional content analysis with Graneheim and Landman approach was used [42]. The transcribed text was read several times and the primary codes were extracted from the data. The 171 primary codes extracted during six sessions, where five members of the research team were present, were analyzed, reviewed, and categorized based on the semantic and conceptual similarities and compacted as far as possible. In this way, 11 subcategories were obtained. Through new interviews, the new codes revealed in the primary encoding process were compared with the existing codes and classified in their subset or in new categories. Through re-comparing of subcategories, four main categories were formed [42].

To increase the research validity, we used the expert check method. Data, concepts, and categories were assessed by three experts to make sure of the correspondence of the classifications with the participants’ statements and, also, of the quality of categorization and relevance of categories. Moreover, the findings were shared with two members of the service providers group, one of them a psychologist and the other a social worker, for their review and the necessary modifications were done based on their viewpoints (member check) [43,44].

## 3 Ethical considerations

The study proposal was approved by the Ethics Committee of the National Institute for Medical Research Development, Deputy for Research, Ministry of Health and Medical Education (Ethics committee approval code: IR. NIMAD.REC.1397.268). All of the interviews in this research have been done with the participants' written informed consent. The interviewers provided participants with information about the research procedure, the confidentiality of information, and non-disclosure of participants' identity and informed them of the research aims and data dissemination plans. Participants’ permission was obtained for recording interviews and they could stop the interview whenever they wanted.

## 4 Findings

This study aimed to answer two questions: 1) How do Iranians' socio-cultural characteristics reveal the relationship between service providers and service receivers, and 2) How do these characteristics affect addiction treatment? The statement of participants on these questions was categorized into five main categories, including non-acceptance of PWUD and drug addiction treatment, mistrust, non-secular treatment, non –acceptance of long-term treatments needing drugs, and inappropriate treatment environments (Table 3). Table 2 summarizes participants’ characteristics.

**Table 2:** Shows demographic data regarding the interviewees such as sex, education, etc.

PWUD		**Service providers**	
*Gender*		*Gender*	
Males	5	Males	5
Female	3	Female	2
*Marital status*		*Education*	
Single	4	Psychology	4
Divorced	4	Consulting	1
*Education*		Social work	1
less than high school	4	High school graduate	1
high school graduate	2	**Policy makers**	
at least some college	2	*Gender*	
*drug use*		Males	4
multi drug	6	Female	0
only opium	2	*Education*	
**Family**		Mechanical engineering	1
*Relationship*		Medical	2
Wives	2	Psychology	1
Mother	2		
Father	1		
Daughter	1		
Sister	1

**Table 3:** The Primary codes, Subcategories, and Main categories.

Primary codes	Subcategories	Main categories
Physical aggression towards PWUD	Mistreatment with PWUD (physical, mental abuse, and torture)	Non-acceptance of PWUD and drug addiction treatment
Very low-quality services		
Disrespectful behavior with the PWUD		
Treating PWUD with harshness and suppression		
The family's feeling of shame	Stigma	
Rejecting behaviors towards PWUD in workplaces		
Accusing women who use drugs of prostitution		
Punishment of stimulating factors		
Dual treatment with PWUD		
Recording data in the Iranian drug abuse treatment information system (IDATIS)	Mistrust among various treatment components	Mistrust
Concerns about the patient's private information abuse		
Concerns about the service providers' financial abuses		
Concerns about some services providers' sexual abuses		
Mistrust between the patients and service providers		
Patients' non-confidence in experts		
Unwillingness to receive psychotherapy services		
Belief in the inefficiency of experts		
More qualifications of peer counselors		
The existence of free parallel treatments	Service providers' profiteering	
Links between service providers' profiteering and patients' addiction		
Low-quality services		
Inappropriate places of treatment		
Inappropriate food in residential centers		
The disproportion between the fee and services		
Abuse of families' status by service providers		
The non-expert relatives' and friends' improper interventions	The confused boundaries between PWUD and khodi (insider)	
PWUD and families' preference to receive help from relatives		
More confidence in relatives and insiders		
Non-specialization of addiction treatment		
More emotional gaps between the PWUD and service providers in hierarchical relations	Treatment rules and indigenous considerations	
Increasing the probability of patients' self-disclosure in equal relationships		
Treatment errors		
PWUD abnormal behaviors as a barrier to treatment	non-secular treatment	
The issuance of permission by God as a need for treatment		
Recognition of God, and commitment to spirituality as a need for treatment		
PWUD multi-consumption of maintenance treatments	Non-acceptance of opioid maintenance treatments	Non-acceptance of long-term treatment needing drugs
Treatment synonymous with abstention		
Using methadone and buprenorphine synonymous with addiction		
Disbelief in maintenance treatment methods		
Impatience with treatment	Short-term orientation in treatment	
Inclination to reach quick results in treatment		
Existence of drug use patterns in the family acceptance of using drugs by the people around	The contexts facilitating drugs abuse	Inappropriate treatment environments
Using the drug as a cultural custom		
Prescription of using substance by physicians		
presence in using drug locations		
Interaction with other PWUD		
Pressure by the people around for using substances		
Volition as the only factor for treatment	The family and community's unrealistic expectations from the treatment and PWUD	
non-acceptance of mistakes and relapse		
PWUD incompetency in sexual relations during the treatment period		
PWUD inability in economic affairs after treatment		

The findings obtained from the interviews were as follows.

## 5 Non-acceptance of PWUD and drug addiction treatment

Non-acceptance of PWUD apparent in their mistreatment and the stigma around using drugs.

### 5.1 Mistreatment with PWUD (physical, mental abuse, and torture)

PWUD experience mistreatment in some treatment centers. For example, one participant described her sister's experience at one treatment center, *“It was a place where they used to beat her. They did very unpleasant behaviors with her, and she didn't want to return. Food provided was nutritionally poor. They did not interact with her appropriately and respectfully, because the* fees *are secured by the government. Non-governmental centers are better and operated with more compassion for PWUD. When I visited a government center, I saw the director screaming at and scolding a new patient.“(Sister, code 19*).

Another PWUD talked about his negative experience in the compulsory treatment center, “My family used to send me to the compulsory camp. I was by the side of someone like (Name of camp manager). The first compulsory camp in Iran. In the end, the mate had an overdose. Suppose! Does anyone bathe ten times in nine months? Is it possible? Is it possible for anyone not to see the sunlight for nine months? Is it possible to have cow's paunch for both lunch and dinner for nine months, the paunch whose dirt stands on the water? The visitors brought confections for the residents of the treatment center but the staff put the confections in front of the dogs. From morning to night, the ration was just a loaf of bread. What did he want to teach me? What did I want to learn there? He did not do anything for me but increase my umbrage at my family.“ (PWUD, code 9).

Some of the participants believed that by this austerity and contempt is meant to convey to the PWUD that their treatment is hard and they should do their best to maintain the treatment. Some of the participants even believed that austerity is necessary.

“They do not give them good food. They are restricted so as not to think the camp is an aunt’s house- ironically meaning a comfortable living place-.” (PWUD, code 21)

### 5.2 Stigma

One of the social barriers, mentioned by participants is the stigma associated with addiction, and its treatment. The families feel ashamed of having one of their members struggling with addiction.

“My family knew my son is a drug user, but they pretended to be ignorant. I also know that there are others in the family who use drugs, and I don't say anything about it. What can one say?” (Mother, code 8).

Of course, this stigma is more prominent if the PWUD is a woman. Women who use drugs are consistently accused of prostitution, which is one of the most important reasons for these women to hide their addiction, which can prevent them from referring to SUD treatment centers.

“If the PWUD is a man, all of the people say he had a mistake, after all, he is a man. But when the PWUD is a woman, they say now that she is addicted, she certainly does other work- they mean prostitution.“(Service provider, code 12)

On the other hand, some people reject the common opinions about the stigma of addiction and the existence of discriminatory behaviors towards the PWUD.

“We can’t deny that the PWUD is not always immoral everywhere. In governmental settings, we sometimes witness attempts to conceal addiction, small robberies, and the continuous snoozing of the PWUD even by their co-workers.“(Policymaker, code 26)

## 6 Mistrust

Mistrust in service providers’ qualifications and proficiency and belief in their profiteering contribute to non-observance of therapeutic advice by the PWUD and, as a result, an unsuccessful professional relationship between therapists and PWUD. This creates reciprocal non-confidence between the two groups. These factors, when combined with the fact that Iranian would trust family and social networks more than institutions and the mismatch between some cultural norms and treatment rules make the treatment conditions difficult.

### 6.1 Mistrust among various treatment parts

Based on the statements of some of the participants it seems that they do not have confidence in each other as the foundations of treatment.

“I don’t trust my patients as I consider the ups and downs of the treatment process and its effects. I can’t count on my patients much. I am not sure their words are honest “(Service provider, code 5)

On the other hand, the service users do not trust the treatment center staff, including service providers, due to their negative experiences. It should be said that the nature of this mistrust in settings specific to maintenance treatment is different from that of mid-term residential centers, which are administered by peer counselors. There are concerns about the founders’ sexual attitudes to the women in mid-term residential centers, as described by family members of the PWUD referred to those centers, described as namous (honor).

“I was in mid-term residential treatment centers. They were seeking either financial misuse or abusing the honor of others. The sponsor took the PWUD’s side for money. These were their criteria. However, they did not admit everyone. For example, they would say Hossein should be my help-seeker because he is rich; his sister is pretty. I do not believe in them at all.” (PWUD, code 10)

It should be mentioned that the statements of this participant subjects such as sexual abuse of PWUD's family members was not his direct experience but he had reached this conclusion through conversation with other clients in mid-term residential centers. In maintenance treatment centers, this distrust results mainly from the concerns about the financial abuses of service providers which will be discussed later.

Another manifestation of this mistrust is the possibility of government abuse of patients' personal information after their registration in the IDATIS system. *“The patients want to receive methadone from maintenance treatment centers, so they should deliver their birth certificates and ID cards. Their names will be entered into the informational system of the Ministry of Health. They want the address and the phone number, too. Instead of these, they refer to the herbalist's shop (the unauthorized treatment centers) and receive their medicines without any trouble.” (Policy maker, code 23)*

“What does IDATIS mean in a country where we are not sure of Iranian APP? The people distrust … However they may claim that our country is secure.“(Policy maker, code 29)

“Some of our clients are the governments' employees, some are from treatment cadre, and some are the students of advanced programs. Well, these people are not willing to provide their information anywhere.” (service provider, code 14)

The statements of some of the participants imply their mistrust of the efficiency of the experts in the SUD treatment process. From their viewpoint, peer counselors are more qualified than professional treatment providers in treatment of SUDs.

“All maintenance treatment centers are alike. They just prescribe nothing more than methadone. There is no difference. One who is not addicted to drugs cannot understand drug addiction. Only rehabilitated individuals can affect the PWUD and motivate others. A psychologist is never of help. The theory is very far from practice. I think one can help those who are themselves addicted. Doctor X or anyone else can't help. Only an addict can understand another addict. The one who is experienced can understand our condition better than a psychologist or a doctor who has just studied theories.“(PWUD, code 9)

“They do not understand what addiction is but they claim they are experts. It takes 5–10 years for one to become either a dermatologist or a hair specialist, but these people (agents of other treatment methods) claim to treat the addicted with their two-week training.” (Policy maker, code 24)

As a result, some treatment seeking PWUD were not very willing to receive counseling and psychotherapy services from professional and certified treatment providers.

### 6.2 Service providers’ profiteering

Some of the participants believed that the staff of treatment centers do not care for the PWUD's wellbeing and are motivated by profits. From this point of view, the ongoing addiction of PWUD supports these centers, so they do not genuinely attempt to rehabilitate the PWUD. It should be mentioned that the idea that the treatment settings seek profit is not specific to settings using opioid maintenance treatment. Some PWUD statements reflect the belief that profiteering drives of mid-term residential treatment centers.

“I pay 3000 Tomans for a medicine which is worth 1000 Toman in there. I needed them, and them, too. You can’t continue your work. If I am not sick and If you are not here, I can’t be cured and find the way.“(PWUD, code 11)

“They ask for money for very trivial things. Instead of food, they give rubbish to our children and ask us to pay the expenses. There is no remedy …. They say: “If you don’t like it, take your child away. They misuse our family’s conditions. It has a vast commercial dimension; they are aware that we don’t have another alternative.“(Father,code 19)

“Some physicians look at their waiting room to find the number of the addicted there. They calculate the money received from the addicted as that day's income.” (Policymaker, code 27)

Moreover, one of the policymakers sees the reason for this view in parallel free treatments across the country.

“Currently, there are non-governmental organizations (NGOs) that work free of charge. We-- the treatment team in maintenance treatment centers--are exposed to the accusation. Therefore, the patient imagines that our profits lie in their addiction. They compare us to the centers providing free-of-charge services.” (Policymaker, code 29)

### 6.3 Treatment rules and indigenous considerations

Some service providers in this study considered adherence to some professional rules as a barrier to treatment. These participants saw keeping distance from the patients as communication between both the service providers and service users. These participants considered non-adherence to those rules as a factor of success for peer counselors.

“There is always a barrier between us and the clients; we cannot act exactly like self-help groups. In self-help groups, the one working with the patient is the guide of the drug user. He is at ease with the patient. But, we, as therapists, depend on our professional rules. Based on our professional rules, one of our faults is getting close to the patients and making friends with them. These are erroneous for us but not for NGOs. It is one of therapy elements for NGOs which is impossible for us.“(Service provider, code 14)

Regarding distance between service providers and PWUD, one of the managers of an NGO active in drug addiction treatment field, who also has psychology training, said, *“All of my co-workers, from the drivers to the butler, are rehabilitated drug addicts. If my driver doesn't see me one day, he cries and says why I can't see Haji or Hajieh (respectively the man and the woman who has performed the pilgrimage to Mecca), because we have effective emotional communication. If I am aware of the characteristics of those who become addicted to drugs, I will treat them both decisively and subtly. “The mother of my children”--he means his wife--plays the role of the good police, and I play the role of the bad one. We are a team. If one makes mistakes two or three times, I will not accept the excuse. Here is not the aunt's house to sleep in free of charge. You are not deserving of our kindness. On the other hand, I will give him another opportunity.“(policymaker, code 26)*

“*The patients are not inclined to self-disclosure. They do not want to talk about the problems in their lives or difficulties with their spouses, especially in group work. I do not dare to talk to my patient about sexual problems, let alone ask about using a condom. This is terrible; you see your patient looking at you with a strange face. This is not my fault, it is something prescribed by the protocol.“(Service provider, code 13)*

### 6.4 The confused boundaries between PWUD and *khodi* (insider)

Improper treatment interventions by unskilled relatives and acquaintances is another barrier that service providers indicated. In some situations, the families, after the denial stage, prefer to receive aid from the extended family. They engage special treatments in more critical stages. Consistent with this, the statements of some participants imply that the PWUD have more confidence in their friends than the service providers and the advice of friends can nullify the therapists’ prescriptions.

“Sometimes, the relatives, based on their insufficient information and the inefficient strategies they provide for the patients, can disturb the treatment. Most of the time, they find fault with the type of treatment and say to the PWUD that the therapy is not sound, and this way, they usually harm the patients.“(Service provider, code 15)

“I asked his cousins for help, after all, they were friends and he listened to them. It was effective. He quitted addiction to the drug but started drinking alcohol. They had asked him to drink alcohol with them instead of using substances.” (Mother, code 20)

“The addicted refer to us and receive their therapy and leave us. Then, they prescribe the same treatment to two of their friends. I have frequently heard this. One of my patients took the syrup and poured water on it. When I asked for the reason, He answered:” One of my friends was in my condition. He used to do this. Now, he has put the drugs aside. I want to do that. They have more confidence in their friends than us. Unfortunately, they trust in their mates rather than us.“(Service provider, code 13)

“One of my friends gave me the feedback that it was better for me to use the drug instead of chemical medicines because the drug is a natural substance. It is better than chemical medicines which are destroying you.“(PWUD, code 11)

## 7 Non-secular treatment

Some participants, stated that the treatment will be completed when the other problematic behaviors of PWUD are put aside. In other words, not using drugs is not sufficient alone for treatment.

“Some ex-addicts propose a wrong way. They say if this is the wrong way, how I was free of drugs for ten years. It has been ten years since you are clean, right, but your way is wrong. Don’t use drugs, but no problem if they ogle! Don’t use drugs, no problem if they steal something from someone! Don’t use drugs and the other thing are not problematic! It is impossible to do everything and do not use drugs. They rob people of their properties, assault, say bad words. If they cannot stay cleaned as they have done these behaviors.“(PWUD, code 4)

“They feel they should not tell lies, or make any mistakes when the start the treatment process. They feel they should be recovered very quickly.“(Service provider, code 12).

According to some participants, recognition of God and spiritualty are the factors that prevent relapse. It is prominent in the statements of some participants that they consider non-treatment of PWUD as the will of God and diminishing misbehavior as the favor of God.

“So far as the disagreeable behaviors such as telling lies and selfishness are not put aside, they return to using drugs when observing someone using drugs or when they are invited. They should be spiritualists. They should have a healthy mind and recognize their God. This prevents them from returning to using drugs.“(Policymaker, code 26)

“I told her mother, this is God’s favor that Mehdi has become addicted to drugs. There is certainly righteousness in it. Its righteousness was that he traveled to Mashhad after 11 years in late 1385 and I asked Imam Reza--one of the religious leaders whose shrine is in Mashhad-- and God to give my son back. At night, I saw in my dreams that a light ray brought my son, Mehdi, and he was healed.“(Father,code 18)

## 8 Non-acceptance of long-term treatments needing drugs

The definition of treatment as based on abstention and the expectation of achieving treatment results in a short time are among the main reasons for reluctance towards maintenance treatment methods.

### 8.1 Non-acceptance of opioid maintenance treatments

The majority of PWUD and their families had a negative view on opioid maintenance treatments and believed that making use of such treatment just leads to polydrug use. Form the viewpoint of some service users, the treatment is equal to abstaining from using drugs and opioid medications.

“I had a methadone maintenance treatment which was useless and I started using two drugs; it became worse. I quit the treatment. My opinion of the clinics changed.“(PWUD, code 2)

“My husband did not accept his son to come to this place. His father says he is not addicted to the pills yet, but he will be addicted soon.” (Mother, code 20)

“I think NA or residential centers are more effective than clinics and hospitals. One cannot get rid of drugs in the clinics because it is just done by pills, methadone, and the like. I tried a methadone treatment, but it was not able to wean the drugs. They prescribed methadone for me which was much more than my daily intake. I do not know what the reason might be, but I was not able to do that in the clinic at all.“(PWUD, code 10)

Traces of such an idea is easily observable among policymakers. “Sometimes, one billion Tomans is spent for three or four addicted being treated. This is while this person is using methadone; he has been able to put drugs aside without methadone and return home, but he has not kicked the drugs and dependence on them. Let me know what the shelter and the philosophy behind it is. Why do they let the addicts whom they keep in shelter be permitted to leave in the morning? They let them live as a mendicant, commit burglary, steal cars, extort money and come back here in the evening. They should commit theft to afford the expenses of the drugs. They sleep there, have food, and, then, they are asked to go out and steal a car. Is this good?“ (Policymaker, code 26).

The interesting point is that participants think that some treatment settings where maintenance medicines are not used have a very unpleasant environment, but they prefer these settings to those that prescribe maintenance medicine. *“The only fault with the setting which does not prescribe maintenance medicines is that they do not have a suitable environment but they admirable in that they do not make use of any medicines and this way the drugs come out of the patients’ bodies. The disadvantage of centers like this is that they use maintenance pills like methadone, which I think is wrong.“(Mother, code 8*).

### 8.2 Short-term orientation in treatment

According to the participants, the treatment should be short-term because long-term maintenance treatments make the PWUD stop.

“When I started my treatment, I thought I will go and will be treated within one or two weeks and come back to my ordinary work and life. When I realized that the change will not happen so quickly, I concluded it is better to use drugs.” (PWUD,code 11)

“I can’t claim that the prolongation of addiction treatment prevents rehabilitation but it makes the patients stop treatment. The patients always ask us, how long is the treatment? How long should we come here?” (Service provider, code 12)

“One of the patients said, I have been coming for one year, why have not I been healed? The families ask the same question. When do they stop taking the medicine? They expect a miracle to happen after one year.” (Service provider, code 6)

“The families continuously say why he/she has not been treated, you are not working properly. They have no patience. They want to reach the desired results very quickly. They think we have a healing elixir or a magic stick which cures everything.“(Service provider, code 14)

This impatience to reach the result is even seen among the families undergoing treatment.

## 9 Inappropriate treatment environments

This is about living in an environment where the factors leading to the use of drugs are abundant and, along with unrealistic expectations of families and even the PWUD themselves, the treatment directs the PWUD to use drugs.

### 9.1 The contexts facilitating drugs abuse

Based on the participants’ statements, some PWUD have grown up or lived in environments where there are no noticeable restrictions as to using drugs and, even, sometimes they are encouraged to use. Sometimes physicians prescribe illicit drugs to treat their patients.

“My wife is my cousin. In our family (originally from Torbat-i Jam) this is very common. There, when you enter a house, you will be entertained first day not by a cup of tea but by some opium. My cousin had grown up there. After years, I say to her, ‘I wish you had prevented me from the first day and did not allow me to use opium (laughing).’” (PWUD, code 3)

“The physician had told my father to use drugs. He had said using some opium would be good. When the doctor gives this suggestion, it is clear that the patients would not follow their treatment. They would say the doctor has prescribed this and quit their treatment.“(PWUD, code 7).

Interaction with other PWUD and peer group pressure, the existence of a model of using drugs among family members, and attendance in places where they have used drugs can create conditions for using drugs. Based on the quotations above, the PWUD's friends are persuading factors for using drugs again due to experiencing social pressure such as mocking and contempt, which lead to using drugs.

As mentioned before, some PWUD and their families saw personal will as the only way of treatment and they considered the role of external factors, such as the environment where the drugs are sold or the interactions with drug-using friends, as a barrier to this decision.

“My friends called me and asked where I was and what I was doing. I answered I did not want to use drugs any longer. They said it did not match me. Get rid of these childish behaviors. All of these are useless. They encouraged me to go to them. I tolerated for six months. During this period I thought of these words. They mocked me and called me incapable. We are having alcoholic liquor. It is not something important. Once I went shopping and saw the clerk using drugs in front of me. I asked him what he was using and he answered …. . As soon as I returned home I closed the door of my room and restarted using drugs.” (PWUD, code 6)

“My son cannot kick drugs due to his friends.“(mother, code 5)

The PWUD participants also reported other people as the main factor for their relapse and even their misbehaviors when drug-using. *“My brother has been the reason for most of my miseries.” (PWUD, code,4*).

Sometimes families relocated in hopes of changing conditions and removing PWUD from the environments that they considered to be a cause of addiction.

“In order to control my son’s conditions, we had non-planed immigration. My husband had a store there which he had rented it. He is now without a job. … My daughter is very nervous. We immigrated in the worst educational and age conditions for her.“(mother, code 8)

## 10 The family and community's unrealistic expectations from the treatment and PWUD

Statements by the participants implied unrealistic expectations of drug addiction treatment and PWUD among both families and community. Most of the participants considered insufficient will as the most prominent factor in SUD treatment, as if the will of PWUD is the only factor for being successful in addiction treatment. Extra emphasis on the will of PWUD leads to ignorance of the role of perseverance and continuation of the treatment process and disability to accept recurrent lapse and relapse by both the family and society. Principally, the participants had unrealistic expectations from SUD treatment programs and did not consider relapse among the ups and downs of the treatment process but perceived it as a barrier in SUD treatment. *“He can go to be treated, that is, if himself wishes, if he has the wish and will, he can solve this problem.“(Wife, code 17*).

One of the participants who had brought his child to a mid-term residential center for the fourth time stated in despair, “If he comes out, he will do the same. We got that out of our system as I know he will relapse. Like before. I always pray not to come out because if he comes out, he will hurt us and the others.“ (Mother, code 8).

Interestingly, the PWUD believed so too. “These damn substances, if you have money but not have the will, you will go willy-nilly, to it, you will consume. But everything refers back to one's will.” (PWUD, code 7).

In this regard, some of the participating families expected that, after a short treatment, the PWUD would perform their familial or spousal duties desirably.

“Most families complain that he/she was better when he/she used the drugs. They expect him/her to change everything and start working immediately, but this does not happen. When the families complain they are actually encouraging PWUD to return to use substances again.“(Service Provider, code 14)

“The ones like my husband are similar to a bone in the throat. It neither goes down nor comes up. It is one problem when they are sick, but when they are clean there are a lot of problems. I told my friends but they did not accept. They said when one is clean, it is not possible to have problems (sexual problems). They saw that their cleanness does not change something; they have problems. I mean they have sexual relations but … when they use substances those relations increase. But when they are clean they cannot have just one perfect sex.” (Wife, code 5)

Unrealistic expectations result in disappointment, distrust in the effectiveness of the treatment, and in some cases, discontinuation of the treatment.

“Why don’t PWUD come? Because they have given up ten times but they have relapsed. One of the problems is that I have given up ten times but I haven’t reached a conclusion.” (service provider, code 23)

## 11 Discussion

This study shows that, in Tehran, cultural and social factors play significant roles in the treatment process. It seems that treatment in Tehran necessarily is not considered a secular intervention with the purpose of abstinence from drugs. In other words, abstaining from drugs is not sufficient for SUD treatment and it is the moralities that, along with abstention, give meaning to the treatment [45,46].

The relationship between treatment and changes in non-addiction behaviors and commitment to morality can be inferred from the statements of participants in the PWUD group, families, policymakers, and some peer counselors. It seems that the experts’ non-emphasis on this connection results from their view of addiction as a disease. The moral principles are so important for these participants that they indicated the behaviors and moral characteristics as a sign of change when evaluating treatment outcomes. This judgmental approach can lead to biases such as discrimination against service users, neglecting their rights, and reduction of treatment standards. This means the PWUD in the treatment process, when they do not show a positive change in their non-addictive behaviors, are less likely to benefit from their families' cooperation and aid and, possibly, do not receive inclusive qualitative services because some service providers are disappointed with their recovery.

In non-secular of treatment, individualistic culture in Iran [47] and emphasis on individual will in resolving problems result in PWUD regarded as people who deserve to be punished. In addition to this, the conservative perspective of Iranians (which is, to some extent, the result of geographic and environmental conditions of the country and the ongoing fear for their endangered survival of life), considers drug use as indulgence of pleasure and irresponsible, thus we observe more intolerance and punitive reactions toward it.

It should be mentioned that punitive behavior with PWUD is a barrier that was reported only by family members and PWUD. Service providers and policymakers were not aware of this punitive behavior or, at least, were not willing to talk about it due to the existing distances between these two groups and the receivers of services. Unrealistic expectations of PWUD by both society and family create little tolerance for mistakes and relapse that are parts of the treatment process (Center for Substance Abuse Treatment, 2006).

The statements of the families participating in this study are full of unrealistic expectations of both treatment and newly-recovered individuals. These expectations were less observed in other groups' statements. It seems that most of these expectations result from the families’ inability to accept that the treatment is a time-consuming process and their desire to reach the expected results in the shortest time possible.

These expectations create stress and fear of failure for the PWUD. As a result of this negative experience, using drugs can act as a relief for PWUD [48]. In addition, treatment follow-up can distinguish between successful and unsuccessful clients in SUD treatment.

The lower acceptance of opioid maintenance treatment was one of the findings of this study that seems to be, to some extent, the result of the Iranian dualistic culture. In Iranian perceptual power with a background of mythological thoughts in the form of lengthy wars and struggles between Iranian and non-Iranian, *Ahuramazda* and *Ahriman*, darkness and lightness, each category is understood against another category [49] and the spaces between the two are not easily understandable. The patients complain that methadone will lead to more severe addiction [50]. The families prefer abstinence-based and high-yield treatment methods; because they perceive medication-based treatment as a less formal form of treatment for PWUD and their families. Thus, the PWUD do not have a medicinal perception of it because they have received it as both medication and the drug. Unlike other groups, none of the service providers attributed the failure of PWUD in treatment to the problems in the treatment method. This can show the service providers' confidence in the efficiency of the treatment method and the presence of some problems in its usage.

There is a lot of stigma attached to methadone [50] and the PWUD who have received it are in doubt of its effectiveness. They consider methadone treatment as a stupid replacement of one kind of drug with another, so, they are distrustful [51] to this kind of treatment and afraid of dependence on it [50].

The contrary to the results of this study, methadone maintenance treatment (MMT) is reported to be a more effective method in some research for reasons such as fear of withdrawal syndrome, having a substitution for the drugs, and keeping one's job, although those individuals did not consider MMT as a satisfactory method for addiction treatment [52].

Non-tolerance of long-term treatment by PWUD receiving services is a barrier that was highly emphasized by service providers. Only one of the PWUD emphasized failure of treatment in the short-term as a barrier to maintenance treatment.

From the perspective of service providers, receivers of services are greatly inclined to quickly achieve positive treatment results. Therefore, for them, living in a mid-term residential center for 21 days is temporally much more preferable than MMT. For these reasons, the service users are highly inclined to yield treatment results as quickly as possible. Katouzyan (2004) believes that Iranians are not inclined to accept prolonged processes most of the time [53]. They do not have the required patience for accumulation and development [54] and prefer temporal benefits to plan for the future [55]. The proverbs such as “tomorrow we will think about tomorrow” or “seize the day” illustrate this approach. The important point is that they ascribe the long processes of SUD treatment to the service providers’ profiteering. Probably for the reason that when the treatment period is predicted to be longer, the receivers of the services are forced to pay more costs for their treatment.

In addition to what was mentioned, some service providers think they are not capable of establishing closer relationships with their clients without compromising their professionality. Following their own professional rules, they are not allowed to make friends with their patients. This, along with the definition of relationship as expert-patient, can operate as a barrier to treatment in Iran. This relationship, in Iranian culture, is associative of relationship with subordinates (hierarchical relationship). In this situation, the ones at the bottom of the hierarchy who are in subordinate positions are always attempting to control the relationship and try to be *zerang* (= adroitness) [56]. The subordinates are powerless in this relationship and feel they are worthless. So, they do whatever they can to conceal this feeling of worthlessness and powerlessness [57]. The manifestation of these attempts is observable in the resistance of the service users against the service providers' treatment interventions [46]. Therefore, in this relationship, gaining one's trust is difficult for both parties. It should not be ignored that in the current atmosphere of Iran's society, the collapse of social cohesion and its consequences has affected this distrust, which increases the fear of intimacy.

Mitchell poses the problem with working with resistant clients in another way. She believes that the more that experts play the role of an expert, they provide more reasons for clients’ resistance [58]. Based on this, more proximity between the therapists and the clients is not meant going beyond boundaries and the professional standards. Rather, therapists are expected to act more flexibly. Another noticeable point is that, based on Iranian cultural features, the acceptance and adherence to the subordinates--here, service providers--necessitates the presence of moralities and dignities [56]. Providing services without any financial expectations is a sign of these moral necessities from the viewpoint of the service users. Because service users suppose the goal of service providers is to gain profits and seek economic benefits, they are suspicious of their sincere help and distrust them.

In this regard, policymakers, considering what they have heard of financial violations at treatment centers and the failure of those centers to achieve expected results, are doubtful about the qualifications and competence of the treatment teams. But the service providers interviewed rarely mentioned service providers’ profiteering. They often thought of PWUD and their families' distrust as a result of conflict between treatment principles and local considerations.

It should be noted that, in Iran, there is a financial relationship between the PWUD and treatment centers and the PWUD receive their medicines directly from these centers. Therefore, physicians adjusting patients' medicines during a long-term treatment process is seen by service users as an attempt to gain more money, which is in contradiction to the seniors’ moral principles. Investigation of other countries' experiences shows that the system can be designed with little or no direct financial relationship between PWUD and service providers [59].

The findings of this study show the lack of a clear boundary between the service users and their families, friends and relatives and their negative interventions in drug addiction treatment. In Iranian culture, the insiders (khodi) and outsiders (gheir-e-khodi) are distinguishable. Being an insider or outsider has a significant effect on Iranians' confidence in each other and clarity. Due to trustfulness and more benevolence, insiders can intervene in one's various problems, including drug treatment. Insiders' improper interventions and the PWUDs' obedience to their prescriptions nullify the experts' roles [60]. Therefore, the low trust by PWUD in service providers can contribute to failing to adhere to providers' treatment advice. It should be noted that in some diseases--especially diseases whose treatment effectiveness are not widely recognized-- what people perceive as important for treatment is not necessarily in accord with evidence-based interventions [61]. In Iran, when treatment advice comes from the individuals who are considered insiders-- especially when the insiders have a similar experience in that disease-- it is more likely to be accepted.

Another theme resulting from this study is the stigma of using drugs and SUD treatment. The participants’ statements on the subject were contradictory. Service providers and policymakers emphasized treatment stigma more than PWUD. Perhaps, part of this sensitivity is the result of the global literature focus on stigma. Some participants considered stigma a barrier to treatment while others do not reproach using drugs, especially using opium; some sub-cultures even advise using it. It should be mentioned that easy access to drugs, such as opium, and the inaccessibility of standard treatment services in some regions are recognized factors in the prevalence of drug use [62].

Perhaps, one of the most important reasons for these contradictory statements is that the Iranian acts are founded on values that are significantly contradictory [56]. Based on this, Iranian society is dualistic to PWUD. In some conditions, the PWUD are rejected, and we observe that they are not employed or fired. However in the same working environments, sometimes co-workers of the PWUD will conceal his/her indiscipline, minor thefts, and frequent napping.

This is not to suggest that addiction is acceptable to those who conceal the misbehavior of PWUD. The matter is that ‘ratting peers out’ is considered immoral act and is not sanctioned by other colleagues, specially that it can get them fired and cause financial loss. Regardless, these cultural complexities greatly affect PWUD’ conditions. There is a great deal of evidence of stigma and its adverse effects on the beginning and success of the treatment [63]. The individuals who have experienced stigma stay longer in residential treatment centers because they have a lower degree of self-efficiency. In addition, they pay more expenses for their treatment [64]. Stigma results in PWUD's frustration with fundamental rights.

Of course, the stigma of addiction to drugs varies depending on the gender, the kind of drug used, and residence [65,66]. Investigation of literary works in Iran implies that many famous Iranian poets have introduced opium as an antidote for the treatment of diseases and, therefore, there is a positive attitude toward using opium in Iranian culture; in some cases, opium is used to control blood pressure, diabetes, blood fat, pain relief, and the like [67]. This evidence shows that there are not many kinds of stigma attached to using some drugs. So, stigma does not have a fixed form in Iranian society and has aligned itself with social dynamics. It seems that these contradictions confront us with more novel barriers than the period when the stigma had a fixed form.

In the end, participants' emphasis on the role of external factors and irresponsibility of PWUD show the service providers' failure to internalize treatment rules and norms that are contrary to their audiences’ cultural qualities, such as fatalism and external control models [54,68]. Based on numerous studies, it is true that the PWUD need to change their social networks to change their conditions [69] but as treatment settings in Iran are not able to form a strong rehabilitation culture, the service users, along with their internalized cultural properties, resort to costly and passive methods such as relocation.

### 11.1 Study limitations

This study had multiple limitations. All study participants were recruited from Tehran, which limits the generalizability of our results to other parts of Iran. Due to the coincidence of the data collection process with the quarantine caused by the Covid-19 pandemic, we faced problems interviewing some individuals.

## 12 Conclusion

Service providers in Iran need to have a better understanding of cultural and social barriers and that influence influencing access and adherence to SUD treatment programs. Awareness of the perceived reasons for acceptance or rejection of some treatment methods, understanding the consequences of the direct financial relationship between service providers and service users and the consequences of hierarchical relationships between some service providers and clients, and awareness of fatalism and styles of external attribution in Iranian culture can assist service providers in planning more appropriate interventions.

## Author contribution statement

Emran Razaghi; Zahra Hooshyari: Performed the experiments; Analyzed and interpreted the data; Contributed reagents, materials, analysis tools or data.

Ali Farhoudian: Conceived and designed the experiments; Performed the experiments; Analyzed and interpreted the data; Contributed reagents, materials, analysis tools or data.

Azam Pilevari: Conceived and designed the experiments; Performed the experiments; Analyzed and interpreted the data; Contributed reagents, materials, analysis tools or data; Wrote the paper.

Alireza noroozi: Conceived and designed the experiments; Performed the experiments; Analyzed and interpreted the data; Wrote the paper.

Ramin Radfar: Analyzed and interpreted the data; Contributed reagents, materials, analysis tools or data; Wrote the paper.

Mohsen Malekinejad: Conceived and designed the experiments; Analyzed and interpreted the data; Wrote the paper.

## Data availability statement

Data will be made available on request.

## Declaration of competing interest

The authors declare that they have no known competing financial interests or personal relationships that could have appeared to influence the work reported in this paper.

## References

[1] Legha R.K., Novins D. (2012). The role of culture in substance abuse treatment programs for American Indian and Alaska Native communities. Psychiatr. Serv..

[2] Weinandy J.T.G., Grubbs J.B. (2021). Religious and spiritual beliefs and attitudes towards addiction and addiction treatment: a scoping review. Addict Behav Rep.

[3] Spooner C., Hetherington K. (2005).

[4] Calsyn D.A., Burlew A.K., Hatch-Maillette M.A., Beadnell B., Wright L., Wilson J. (2013). An HIV prevention intervention for ethnically diverse men in substance abuse treatment: pilot study findings. Am. J. Publ. Health.

[5] Paris M., Silva M., Añez-Nava L., Jaramillo Y., Kiluk B.D., Gordon M.A., Nich C., Frankforter T., Devore K., Ball S.A. (2018). Culturally adapted, web-based cognitive behavioral therapy for Spanish-speaking individuals with substance use disorders: a randomized clinical trial. Am. J. Publ. Health.

[6] Burlew A.K., Copeland V.C., Ahuama-Jonas C., Calsyn D.A. (2013). Does cultural adaptation have a role in substance abuse treatment?. Soc. Work. Publ. Health.

[7] Campbell C.I., Wells R., Alexander J.A., Jiang L., Nahra T.A., Lemak C.H. (2007). Tailoring of outpatient substance abuse treatment to women, 1995-2005. Med. Care.

[8] Burlew A.K., McCuistian C., Lanaway D., Hatch-Maillette M., Shambley-Ebron D. (2020). One size does not fit all: a NIDA CTN inspired model for community engaged cultural adaptation. J. Subst. Abuse Treat..

[9] Guerrero E., Andrews C.M. (2011). Cultural competence in outpatient substance abuse treatment: measurement and relationship to wait time and retention. Drug Alcohol Depend..

[10] Matthews S. (2019). The Stigma of Addiction: an Essential Guide.

[11] Matthews S., Dwyer R., Snoek A. (2017). Stigma and self-stigma in addiction. J. bioeth. Inq..

[12] Moscati A., Mezuk B. (2014). Losing faith and finding religion: religiosity over the life course and substance use and abuse. Drug Alcohol Depend..

[13] Grim B.J., Grim M.E. (2019). Belief, behavior, and belonging: how faith is indispensable in preventing and recovering from substance abuse. J. Relig. Health.

[14] Saloner B., Cook B.L. (2013). Blacks and Hispanics are less likely than whites to complete addiction treatment, largely due to socioeconomic factors. Health Aff..

[15] Niv N., Pham R., Hser Y.-I. (2009). Racial and ethnic differences in substance abuse service needs, utilization, and outcomes in California. Psychiatr. Serv..

[16] Pinedo M., Zemore S., Rogers S. (2018). Understanding barriers to specialty substance abuse treatment among Latinos. J. Subst. Abuse Treat..

[17] Daniewicz S. (2014).

[18] Evans J.S. (2006).

[19] Westermeyer J. (1987). Cultural patterns of drug and alcohol use: an analysis of host and agent in the cultural environment. Bull. Narc..

[20] Barnett A.I., Hall W., Fry C.L., Dilkes‐Frayne E., Carter A. (2018). Drug and alcohol treatment providers' views about the disease model of addiction and its impact on clinical practice: a systematic review. Drug Alcohol Rev..

[21] Edland-Gryt M., Skatvedt A.H. (2013). Thresholds in a low-threshold setting: an empirical study of barriers in a centre for people with drug problems and mental health disorders. Int. J. Drug Pol..

[22] Whetten K., Leserman J., Whetten R., Ostermann J., Thielman N., Swartz M., Stangl D. (2006). Exploring lack of trust in care providers and the government as a barrier to health service use. Am. J. Publ. Health.

[23] Jauffret-Roustide M., Cohen J., Poisot-Martin I., Spire B., Gossop M., Carrieri M.P., Group M.S. (2012). Distributive sharing among HIV–HCV co-infected injecting drug users: the preventive role of trust in one's physician. AIDS Care.

[24] Lago R.R., Peter E., Bógus C.M. (2017). Harm reduction and tensions in trust and distrust in a mental health service: a qualitative approach. Subst. Abuse Treat. Prev. Pol..

[25] Harris M., Rhodes T., Martin A. (2013). Taming systems to create enabling environments for HCV treatment: negotiating trust in the drug and alcohol setting. Soc. Sci. Med..

[26] Cohn L., Esparza del Villar O. (2015). https://bit.ly/2sAF5Iw.

[27] Ja D.Y., Aoki B. (1993). Substance abuse treatment: culture and barriers in the Asian-American community. J. Psychoact. Drugs.

[28] Riper H., Blankers M., Hadiwijaya H., Cunningham J., Clarke S., Wiers R., Ebert D., Cuijpers P. (2014). Effectiveness of guided and unguided low-intensity internet interventions for adult alcohol misuse: a meta-analysis. PLoS One.

[29] Verissimo A.D.O., Grella C.E. (2017). Influence of gender and race/ethnicity on perceived barriers to help-seeking for alcohol or drug problems. J. Subst. Abuse Treat..

[30] Guerrero E.G., Marsh J.C., Cao D., Shin H.-C., Andrews C. (2014). Gender disparities in utilization and outcome of comprehensive substance abuse treatment among racial/ethnic groups. J. Subst. Abuse Treat..

[31] Witbrodt J., Kaskutas L.A., Grella C.E. (2015). How do recovery definitions distinguish recovering individuals? Five typologies. Drug Alcohol Depend..

[32] Volkow N.D. (2020). Stigma and the toll of addiction. N Engl J Med Overseas Ed.

[33] Ronzani T.M., Higgins-Biddle J., Furtado E.F. (2009). Stigmatization of alcohol and other drug users by primary care providers in Southeast Brazil. Soc. Sci. Med..

[34] Tabrizi A.M., Ghaderi S. (2011). Norms facilitating drug abuse in Iranian ethnic sub-cultures. Soc. Probl..

[35] Amiri R.S., abdolmaleki H. (2015). Pathological approach to the cultural strategies for prevention from the addiction to drugs in Iran, from policymaking to operationalization. Soc Cult Res J.

[36] Hajli A., Zakariaey M.A., Kermani S.H. (2010). Iranians' attitude towards drug abuse. Soc. Probl. Iran..

[37] Nyashanu T., Visser M. (2022). Treatment barriers among young adults living with a substance use disorder in Tshwane, South Africa. Subst. Abuse Treat. Prev. Pol..

[38] Cameron A., Abrahams H., Morgan K., Williamson E., Henry L. (2016). From pillar to post: homeless women's experiences of social care. Health Soc. Care Community.

[39] Pauly B.B. (2008). Shifting moral values to enhance access to health care: harm reduction as a context for ethical nursing practice. Int. J. Drug Pol..

[40] Jørgensen U. (2001). Grounded theory: methodology and theory construction. Int. Encycl. Soc. Behav. Sci..

[41] Sadeghi R., Zanjari N. (2017). The inequality of development in the 22 districts of Tehran metropolis. Soc. Welfare.

[42] Graneheim U.H., Lundman B. (2004). Qualitative content analysis in nursing research: concepts, procedures and measures to achieve trustworthiness. Nurse Educ. Today.

[43] Hancock B., Ockleford E., Windridge K. (2001).

[44] Flick U. (2022).

[45] Galanter M., White W.L., Hunter B.D. (2019). Cross-cultural applicability of the 12-Step model: a comparison of Narcotics Anonymous in the USA and Iran. J. Addiction Med..

[46] Levin J.D. (1977).

[47] Asghari S.A. (2020). Conceptual analysis of individualism in Iran and its relation with classical concepts of individualism. J. Sociol. soc. Inst..

[48] Yu J., Clark L.P., Chandra L., Dias A., Lai T.-F.M. (2009). Reducing cultural barriers to substance abuse treatment among Asian Americans: a case study in New York City. J. Subst. Abuse Treat..

[49] Rashed M.A., Tahami M. (2009). The evolution of Iranian mythology based on prehistoric and Kiani myths. Int. J. Persian Lit..

[50] Khazaee-Pool M., Moeeni M., Ponnet K., Fallahi A., Jahangiri L., Pashaei T. (2018). Perceived barriers to methadone maintenance treatment among Iranian opioid users. Int. J. Equity Health.

[51] Bobrova N., Rhodes T., Power R., Alcorn R., Neifeld E., Krasiukov N., Latyshevskaia N., Maksimova S. (2006). Barriers to accessing drug treatment in Russia: a qualitative study among injecting drug users in two cities. Drug Alcohol Depend..

[52] Shamsalinia A., Norouzi K., Fallahi-Khoshknab M., Farhoudian A., Ghaffari F. (2017). Experiences of substance abusers from methadone maintenance therapy. Med. J. Islam. Repub. Iran.

[53] Katouzian H. (2004). The short-term society: a study in the problems of long-term political and economic development in Iran. Middle E. Stud..

[54] Moradi H.G. (2007).

[55] Hosseinpanahi H., Baizidi R. (2020). Culture and development: comparison of cultural development indicators in Japan, britain and Iran. J Econ Soc Dev.

[56] Beeman W.O. (1986).

[57] McClelland D.C., Wanner E., Davis W.N., Kalin R. (1972).

[58] Mitchell C.W. (2009).

[59] Seyler T., Giraudon I., Noor A., Mounteney J., Griffiths P. (2021). Is Europe facing an opioid epidemic: what does European monitoring data tell us?. Eur J Pain Suppl.

[60] Pilevari A., Asl M.Z. (2021). The effects of drug addiction treatment methods on families' behaviors: the Congress 60 treatment method. J. Subst. Abuse Treat..

[61] Walker H.K., Hall W.D., Hurst J.W. (1990).

[62] McLaughlin G.T. (1975). The poppy is not and ordinary flower: a survey of drug policy in Iran. Fordham Law Rev..

[63] Cernasev A., Hohmeier K.C., Frederick K., Jasmin H., Gatwood J. (2021). A systematic literature review of patient perspectives of barriers and facilitators to access, adherence, stigma, and persistence to treatment for substance use disorder. Explor Res Clin Soc Pharm.

[64] Luoma J.B., Kulesza M., Hayes S.C., Kohlenberg B., Larimer M. (2014). Stigma predicts residential treatment length for substance use disorder. Am. J. Drug Alcohol Abuse.

[65] Etesam F., Assarian F., Hosseini H., Ghoreishi F.S. (2014). Stigma and its determinants among male drug dependents receiving methadone maintenance treatment. Arch. Iran. Med..

[66] Mokri A. (2002). Brief overview of the state of drug abuse in Iran. Arch. Iran. Med..

[67] Zarghami M. (2015). Iranian common attitude toward opium consumption. Iran J Psychiatry Behav Sci.

[68] Farasatkhah M. (2015). We Iranians : Nashreney.

[69] Pettersen H., Landheim A., Skeie I., Biong S., Brodahl M., Oute J., Davidson L. (2019). How social relationships influence substance use disorder recovery: a collaborative narrative study. Subst. Abuse.

